# Supplementary figures and images for: The Arabidopsis active demethylase ROS1 cis-regulates defence genes by erasing DNA methylation at promoter-regulatory regions
Source: eLife. 2021 Jan 20;10:e62994. doi: 10.7554/eLife.62994 (PMC7880685; doi:10.7554/eLife.62994)

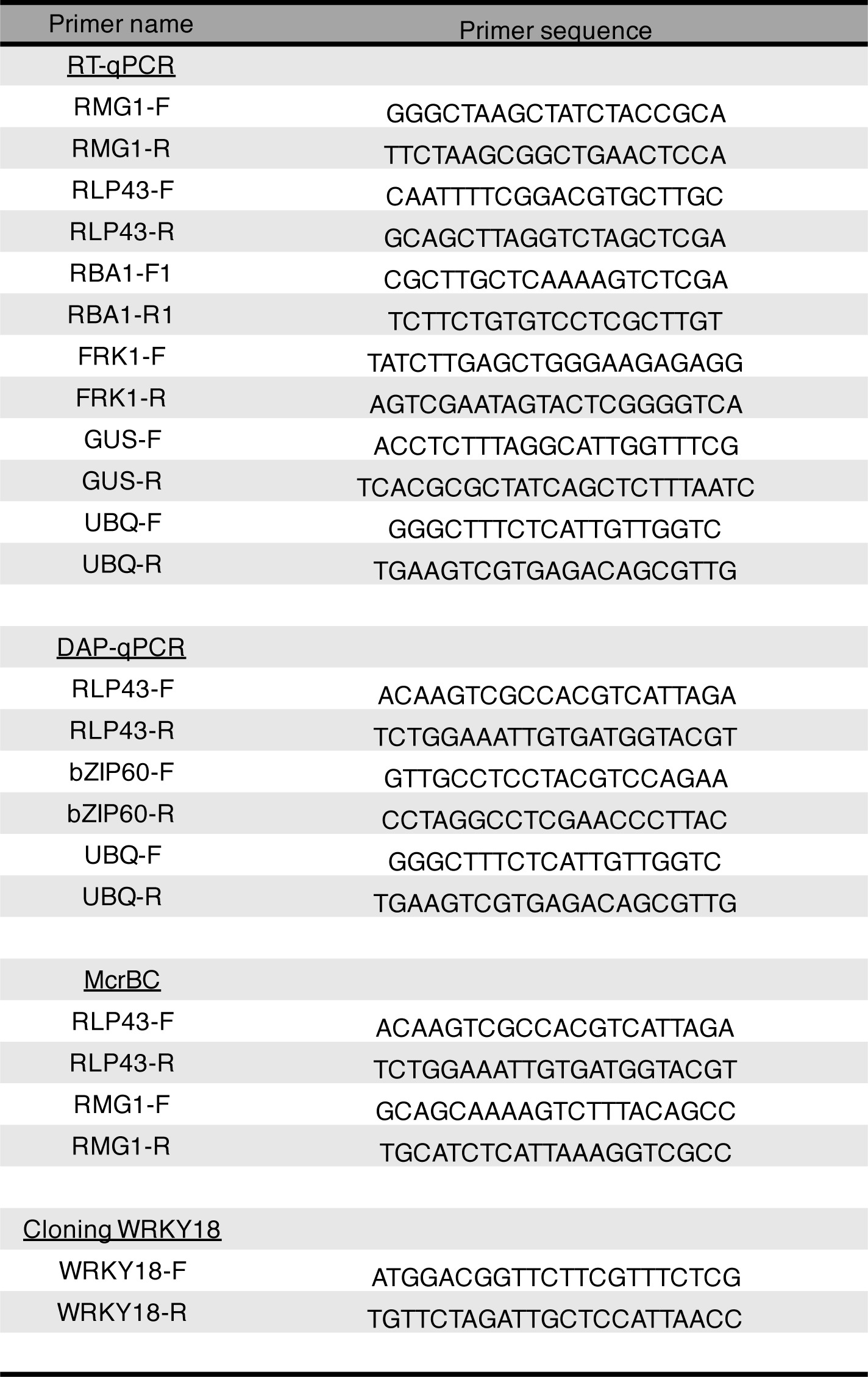

Supplement: Supplementary file 2. [file elife-62994-supp2.jpg]
