# Supplementary material for: The Arabidopsis active demethylase ROS1 cis-regulates defence genes by erasing DNA methylation at promoter-regulatory regions
Source: eLife. 2021 Jan 20;10:e62994. doi: 10.7554/eLife.62994 (PMC7880685; doi:10.7554/eLife.62994)
Supplement: Transparent reporting form [file elife-62994-transrepform.docx]

***eLife’s* transparent reporting form**

We encourage authors to provide detailed information *within their submission* to facilitate the interpretation and replication of experiments. Authors can upload supporting documentation to indicate the use of appropriate reporting guidelines for health-related research (see [EQUATOR Network](http://www.equator-network.org/%20)), life science research (see the [BioSharing Information Resource](https://biosharing.org/)), or the [ARRIVE guidelines](http://www.plosbiology.org/article/info:doi/10.1371/journal.pbio.1000412) for reporting work involving animal research. Where applicable, authors should refer to any relevant reporting standards documents in this form.

If you have any questions, please consult our Journal Policies and/or contact us: [editorial@elifesciences.org](mailto:editorial@elifesciences.org).

**Sample-size estimation**

- You should state whether an appropriate sample size was computed when the study was being designed
- You should state the statistical method of sample size computation and any required assumptions
- If no explicit power analysis was used, you should describe how you decided what sample (replicate) size (number) to use

Please outline where this information can be found within the submission (e.g., sections or figure legends), or explain why this information doesn’t apply to your submission:

Sample sizes are indicated in the figure legends and/or the Material and Method section.

**Replicates**

- You should report how often each experiment was performed
- You should include a definition of biological versus technical replication
- The data obtained should be provided and sufficient information should be provided to indicate the number of independent biological and/or technical replicates
- If you encountered any outliers, you should describe how these were handled
- Criteria for exclusion/inclusion of data should be clearly stated
- High-throughput sequence data should be uploaded before submission, with a private link for reviewers provided (these are available from both GEO and ArrayExpress)

Please outline where this information can be found within the submission (e.g., sections or figure legends), or explain why this information doesn’t apply to your submission:

Detailed information regarding replicates are indicated in the figure legends and/or the Material and Method section. High-throughput small RNA sequencing and bisulfite sequencing datasets are accessible on SRA under the bioproject accession: PRJNA434451.

***Pto* DC3000 apoplastic growth**: At least 6 biological replicates were collected per experiment. The values from three independent experiments were considered for the comparative analysis.

***Pto* DC3000 vascular propagation**: At least 10 biological replicates were collected per experiment. The values from three independent experiments were considered for the comparative analysis.

**qRT-PCR**: For each condition, two technical and three biological replicates (2 leaves per individual plant per condition) were used. Two independent experiments were performed.

**DAP-qPCR**: For each experiment, one biological replicate (DNA was extracted from leaves of 3 independent plants) per condition has been quantified. Two independent experiments were performed.

**Preparation of samples for sRNA-seq and Bs-seq experiments**:

5-week-old leaves from three individual plants of each genotype were used for small RNA deep-sequencing (two independent biological replicates for each analysis).

**Northern blot analysis**:

5-week-old leaves from three individual plants of each genotype were used for the low molecular weight Northern blot analysis.

We excluded one qRT-PCR sample (*ros1dcl23* mock replicate 3) from one experiment shown in figure 3G (*AT1G08940* and *RLP43*) and figure 2-figure supplement 3 (*RMG1*) due to a technical problem during the RNA extraction or to RNA degradation.

**Statistical reporting**

- Statistical analysis methods should be described and justified
- Raw data should be presented in figures whenever informative to do so (typically when N per group is less than 10)
- For each experiment, you should identify the statistical tests used, exact values of N, definitions of center, methods of multiple test correction, and dispersion and precision measures (e.g., mean, median, SD, SEM, confidence intervals; and, for the major substantive results, a measure of effect size (e.g., Pearson's r, Cohen's d)
- Report exact p-values wherever possible alongside the summary statistics and 95% confidence intervals. These should be reported for all key questions and not only when the p-value is less than 0.05.

Please outline where this information can be found within the submission (e.g., sections or figure legends), or explain why this information doesn’t apply to your submission:

For each experiment and analysis, information about statistical tests are detailed in each relevant figure legend.

(For large datasets, or papers with a very large number of statistical tests, you may upload a single table file with tests, Ns, etc., with reference to sections in the manuscript.)

**Group allocation**

- Indicate how samples were allocated into experimental groups (in the case of clinical studies, please specify allocation to treatment method); if randomization was used, please also state if restricted randomization was applied
- Indicate if masking was used during group allocation, data collection and/or data analysis

Please outline where this information can be found within the submission (e.g., sections or figure legends), or explain why this information doesn’t apply to your submission:

Samples were not allocated into experimental groups, so this information does not apply to our study.

**Additional data files (“source data”)**

- We encourage you to upload relevant additional data files, such as numerical data that are represented as a graph in a figure, or as a summary table
- Where provided, these should be in the most useful format, and they can be uploaded as “Source data” files linked to a main figure or table
- Include model definition files including the full list of parameters used
- Include code used for data analysis (e.g., R, MatLab)
- Avoid stating that data files are “available upon request”

Please indicate the figures or tables for which source data files have been provided:

We uploaded 10 additional source data files as described below:

Figure 1-source data 1. Original data of bacterial propagation assays for figure 1A, C, D and F

Figure 2-source data 1. Original qRT-PCR data for figure 2A and F, and bacterial propagation data for figure 2B-C

Figure 2-source data 2. Original qRT-PCR data for figure 2-figure supplement 3

Figure 3-source data 1. Original qRT-PCR data for figure 3G

Figure 3-source data 2. Original transcriptomic data used for the heatmap presented in figure 3B

Figure 4-source data 1. Original qRT-PCR data of GUS transcript quantification for figure 4E

Figure 5-source data 1. Original DAP-qPCR data for figure 5B

Figure 5-source data 2. Original DAP-qPCR data for figure 5-figure supplement 1

Figure 6-source data 1. Original McrBC-qPCR, qRT-PCR and bacterial propagation data for figure 6B-E

Figure 6-source data 2. Original qRT-PCR data for figure 6-figure supplement 1
